# Microstructure Evolution Control and Performance Degradation of SA-178 Grade C Boiler Tubes Driven by Pearlite Spheroidization

**DOI:** 10.3390/ma19020270

**Published:** 2026-01-09

**Authors:** Adimas Aprilio Hardinanto, Anne Zulfia Syahrial, Amin Suhadi, Eka Febriyanti, Gilang Cempaka Kusuma, Hamdani, Ridwan, Andon Insani, Muhammad Refai Muslih, Bharoto, Sairun, Suryadi

**Affiliations:** 1 Department of Metallurgical and Materials Engineering, Faculty of Technology, University of Indonesia, Jl. Lingkar Pondok Cina, Depok 16424, Indonesia; adimas.aprilio31@ui.ac.id (A.A.H.); anne.zulfia@ui.ac.id (A.Z.S.); 2 Research Center for Structural Strength Technology, National Research and Innovation Agency, Gedung 220 KST BJ.Habibie, Tangsel 15314, Indonesia; ekaf002@brin.go.id (E.F.); gila001@brin.go.id (G.C.K.); hamd001@brin.go.id (H.); 3 Research Center for Radiation Detection and Nuclear Analysis Technology, KST BJ.Habibie, Tangsel 15314, Indonesia; ridw001@brin.go.id (R.); andon.insani@brin.go.id (A.I.); mref001@brin.go.id (M.R.M.); bharoto@brin.go.id (B.); sairun@brin.go.id (S.); 4 Research Center for Advanced Material, KST BJ.Habibie, Tangsel 15314, Indonesia; sury011@brin.go.id

**Keywords:** boiler, tube, spheroidized, pearlite, neutron

## Abstract

SA 178 grade C carbon steel is a material commonly used in boiler tubes. Boilers are crucial in the energy industry; however, their service life degrades over time. If a boiler malfunctions, processing operations must be halted, resulting in financial losses for the company. The aim of this study is to examine the effect of microstructural evolution, especially the transformation of lamellar pearlite into spheroidized pearlite, on the service life degradation of boiler tubes. Understanding these changes is essential for preventing catastrophic system failures. The methodology involves the use of Small-Angle X-ray Scattering (SAXS) supported by metallographic analysis, Scanning Electron Microscopy (SEM), Energy-Dispersive X-ray (EDX) Spectroscopy, and mechanical testing. The SAXS results indicate that the microstructure of SA 178, which initially consisted of ferrite and lamellar pearlite, gradually transforms into spheroidized pearlite. These microstructural changes lead to reductions in tensile strength from 523 MPa for 0% spheroidization to 335 MPa for 100% spheroidization, as well as a reduction in hardness from 175 HV to 89 HV, ultimately decreasing the service life of the boiler tube.

## 1. Introduction

The equipment employed in most Indonesian oil and gas industries has been operating for 20 to 30 years, and is therefore nearing the end of its manufacturer-recommended lifetime; this is especially true for equipment that runs for 24 h a day at elevated temperatures and static loads. Thus, the oil and gas industry must accurately assess the current remaining useful life and integrity of this equipment to prevent catastrophic failure, which could cause damage and fatality. An example of immediate damage is the cessation of production; however, preventing indirect damage is as crucial as preventing immediate damage, because it could affect national economic stability by causing fuel shortages and electricity blackouts across the region [1,2]. Another minor effect of damage is the loss of consumer trust in companies affiliated with the oil and gas industry. In general, the degradation of material properties in industrial equipment such as boiler tubes can be caused by corrosion, erosion, mechanical damage, overpressure, and microstructural evolution. However, corrosion, erosion, and mechanical damage are visually apparent, whereas changes in microstructure cannot be directly observed by visual inspection. Industrial equipment operated at elevated temperatures while carrying static loads, such as boilers, will undergo gradual microstructural evolution over time, degrading its mechanical properties and thus reducing its remaining useful life; therefore, preventive maintenance should be carried out to avoid catastrophic damage by correctly assessing the equipment’s remaining useful life [3,4]. During maintenance of power plant boiler components, the remaining useful life of steel materials is commonly estimated through a combination of non-destructive testing (NDT) and hardness testing. Hardness reflects not only a material’s resistance to deformation, but also the extent of long-term degradation. Therefore, the remaining service life of a material can be estimated from changes in hardness data when the operating temperature and pressure are known. However, microstructural inhomogeneity in different parts of the material may lead to anomalous hardness values. To accurately characterize material degradation, both hardness measurements and microstructural observations should be considered. Zhao Q. et al. [5] provide crucial insights into the microstructural evolution of spheroidized pearlite, thereby providing a better understanding of its mechanical properties and potential applications in various industrial fields. The spheroidizing process aims to transform carbide particles in steel from irregular, elongated lamellar morphologies into a rounded (spheroidal) form. This significantly alters their size and distribution, thereby enhancing the material’s ductility and mechanical strength. Therefore, research is needed on the mechanism of SA 178 boiler tube service life degradation caused by microstructural evolution. A complete and integrated study investigating the relationship between the degree of spheroidization and mechanical property degradation was conducted using Small-Angle X-ray Scattering (SAXS), which enables detailed elucidation of the transformation mechanism from lamellar to spheroidized pearlite. Further supporting tests should be conducted to obtain comprehensive data on the remaining useful life, allowing manufacturers to make timely decisions to prevent damage and minimize losses [5].

## 2. Materials and Methods

### 2.1. Materials

The material selected for this research was AISI SA 178 grade C medium-carbon steel, which is commonly used in boiler pipes. Its composition is listed in Table 1. The hardness of untreated ASME SA 178 is 175 HV, while its tensile strength is about 325 MPa, and its yield strength is about 180 MPa. The steel was obtained in the form of a round bar to make it easier for the tensile test specimen to be machined and heat-treated [6].

### 2.2. Methods

The methodology of this study involved the use of Small-Angle X-ray Scattering (SAXS), supported by metallographic analysis, Scanning Electron Microscopy (SEM), Energy-Dispersive X-ray (EDX) Spectroscopy, and mechanical testing. To obtain spheroidized pearlite at different percentages, the specimens were heat-treated at various temperatures and holding times, and their microstructure, particle size distribution, and percentage of spheroidized pearlite were evaluated [7]. The parameters of the heat treatment are listed in Table 2.

Scanning Electron Microscopy (SEM) was applied to investigate microstructural changes and to measure the spheroidization ratios (SRs) of the specimens. To measure the spheroidization ratios, the SEM micrographs were analyzed using the image analysis program Image J version 1.5.4. This program was also used to obtain local information regarding the area and the aspect ratio of the cementite particles. Referring to Ho Seon Joo et al. [8], the spheroidized cementite particles were considered to be an elliptical shape with an aspect ratio represented by the ratio of the major to minor axes. The cementite was considered to be spheroidized up to an aspect ratio of 5:1. This ratio is calculated based on a uniform metallographic criterion. The cementite area was used to calculate the spheroidization ratio (SR), and Equation (1) was used for experimental measurements.

(1) $$\mathrm{Spheroidization}\;\mathrm{ratio}\;(\%)=\frac{Vs}{Vt}\times 100$$

Here, Vt and Vs are the total and spheroidized cementite area, respectively. The circularity of the cementite was calculated using Equation (2).

(2) $$\mathrm{Circularity}=\frac{A_{s}}{{P_{s}}^{2}}\times 4\pi$$

Here, A_s_ is the spheroidized cementite area, and P_s_ is the perimeter of cementite particles. The result of this calculation was then used as a basis for testing mechanical properties and neutron scattering. A neutron diffractometer was employed using the neutron diffraction method to characterize crystal structure, texture, and residual stress. A texture neutron diffractometer (DN2) was installed in the experimental hall of the reactor (XHR) on beam tube S5 and set to a neutron wavelength of 1.2799 angstroms using a Helium monitor detector and a BF main detector. Material characterization was conducted at a reactor power of 5 MW using the preset count (PRSC) method. Since the reactor operated only at 5 MW, it was challenging to obtain a complete pole figure through texture characterization, as a minimum power of 15 MW was required. For texture characterization, the sample was placed on an Euler cradle located on the sample table. The sample was then tilted and rotated alternately within a specific angular range. Tilting was performed within the angle range of 0° ≤ χ ≤ 90°, while rotation was carried out within the angle range of 0° ≤ φ ≤ 360°. Moreover, to study the evolution from lamellar pearlite to spheroidized pearlite, Small-Angle X-ray Scattering (SAXS) was employed. To assess the mechanical properties, a hot tensile test and a hardness test were conducted. Finally, pearlite spheroidization was examined via optical metallography and scanning electron microscopy, and Energy-Dispersive X-ray (EDX) Spectrometry was performed to examine the particle composition.

## 3. Results and Analysis

The results of the experiments conducted in this study provide valuable information that can guide further discussion.

### 3.1. Chemical Composition

The chemical composition test result indicated that the specimen composition is in accordance with the AISI SA 178 grade C specification, and no impurities were found in the specimens. Details of the chemical composition test can be seen in Table 1. The carbon content is lower (0.18%) than the standard maximum of 0.35%, which means the amount of cementite in the pearlite is lower than the standard specification. A reduction in carbon content below the specified range for SA-178 Grade C systematically decreases the pearlite volume fraction, resulting in a higher proportion of ferrite. This reduction in cementite content promotes a faster spheroidization response due to easier fragmentation of pearlitic lamellae, while also leading to a lower final carbide population.

### 3.2. Results of Metallographic Examination

**Figure 1:**
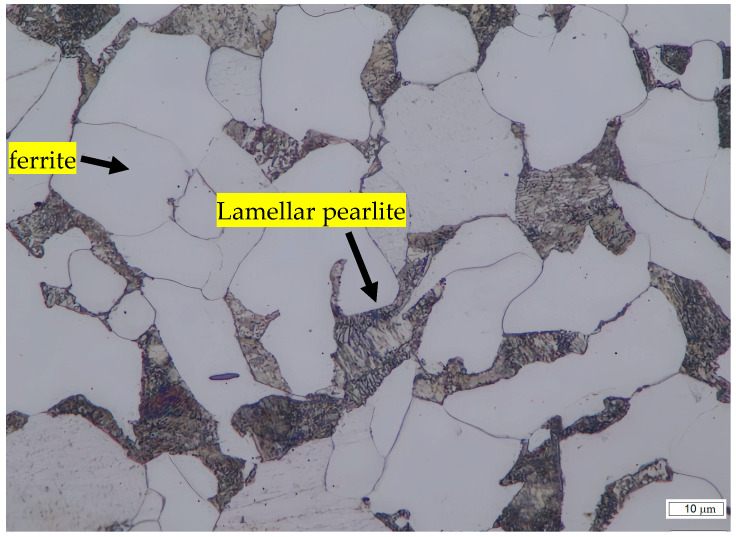
Microstructure of untreated specimen consists of a mixture of ferrite and lamellar pearlite.

**Figure 2:**
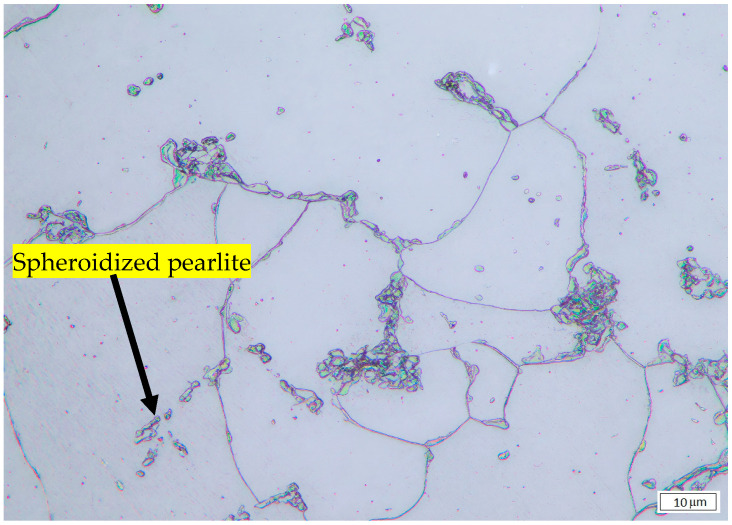
Microstructure of specimen heat-treated at 750 °C for 30 h. Most of the lamellar pearlite was converted to spheroidized pearlite.

**Figure 3:**
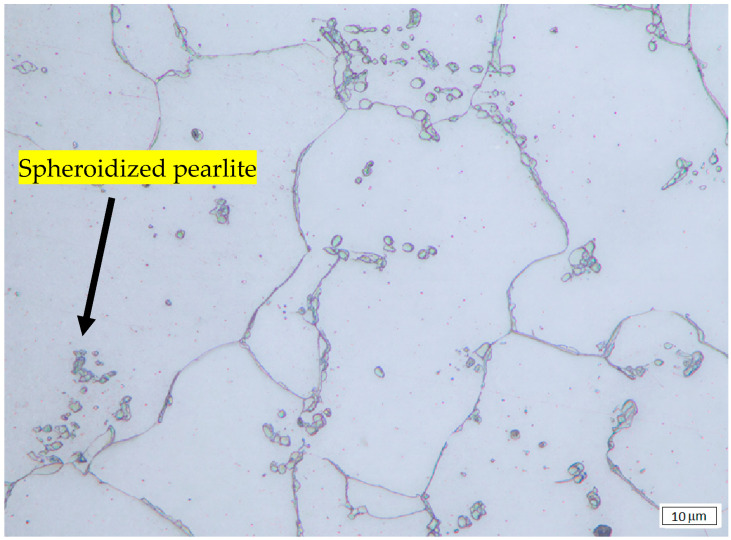
Microstructure of the specimen heat-treated at 650 °C for 72 h. The lamellar pearlite is completely converted to spheroidized pearlite.

The microstructure of untreated and heat-treated samples at various times and temperatures was examined by optical microscopy. The results are presented in Figure 1, Figure 2 and Figure 3. Figure 1 shows that for untreated SA 178 specimens, the microstructure consists of a mixture of ferrite and lamellar pearlite. However, after several hours of holding time at high temperature, the lamellar pearlite becomes spheroidized [9,10,11] (Figure 2 and Figure 3).

### 3.3. Scanning Electron Microscopy (SEM) Results

**Figure 4:**
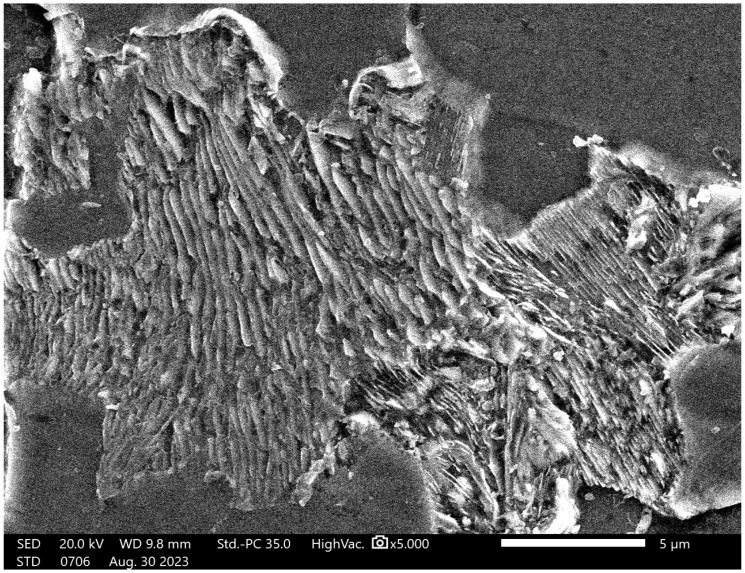
Microstructure of untreated specimen shows that pearlite has lamellar structure.

**Figure 5:**
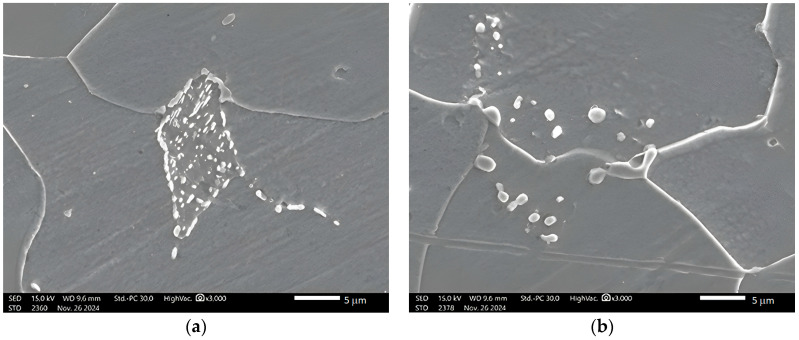
(**a**) SEM result of specimen heat-treated at 750 °C for 30 h. Most of the lamellar pearlite is converted to spheroidized pearlite. (**b**) SEM of specimen heat-treated at 650 °C for 72 h. The lamellar pearlite is completely converted to spheroidized pearlite.

The results of the SEM examination of the microstructure of untreated and heat-treated samples at different temperatures and holding times can be seen in Figure 4 and Figure 5. It is shown that pearlite initially has lamellar cementite (Figure 4), and after heat treatment, the lamellar pearlite is transformed to spheroidized pearlite, indicating that the cementite has become spheroidized (Figure 5).

### 3.4. Energy-Dispersive X-Ray (EDX) Spectrometry

**Figure 6:**
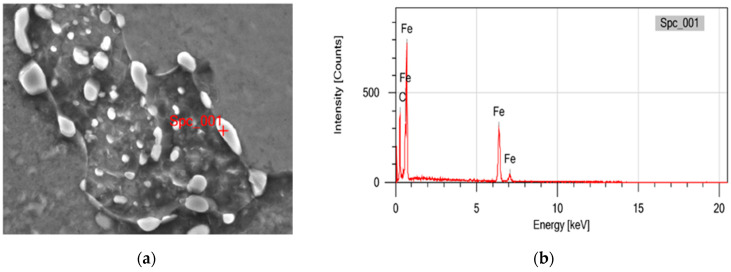
(**a**) EDX test location on particles that formed after heat treatment. (**b**) EDX spectrum of particles, which are suspected to be spheroidized cementite.

To identify the kinds of particles formed after heat treatment, Energy-Dispersive X-ray (EDX) Spectrometry was conducted on samples heated for specific durations and at different temperatures, as shown in Figure 6 and Table 3. The spectrum observed in spheroidized pearlite is dominated by Fe and C, confirming that the particles consist of Fe_3_C. This indicates that the spheroidized particles originate from the transformation of lamellar Fe_3_C, which initially alternated with ferrite in the pearlite structure. No other spectra appeared in the EDX analysis for these particles.

Figure 6 and Table 3 indicate that the dominant elements are Fe and C, which confirms that the particles are spheroidized cementite (Fe_3_C) resulting from pearlite spheroidization after heat treatment.

### 3.5. Spheroidization Ratios Obtained by Image J

**Figure 7:**
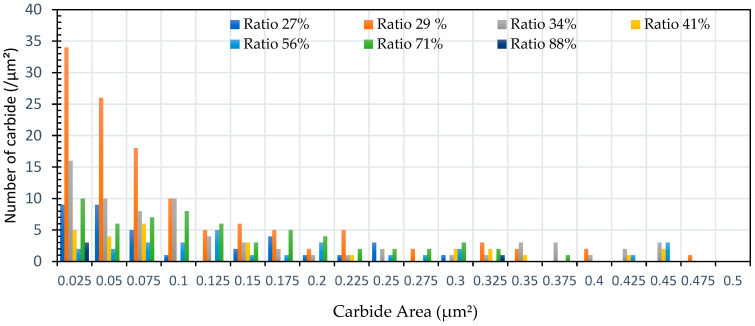
The distribution of carbide quantity (per µm^2^) vs. carbide area (µm^2^) for specimens subjected to heat treatment with varying spheroidization ratios (%).

**Figure 8:**
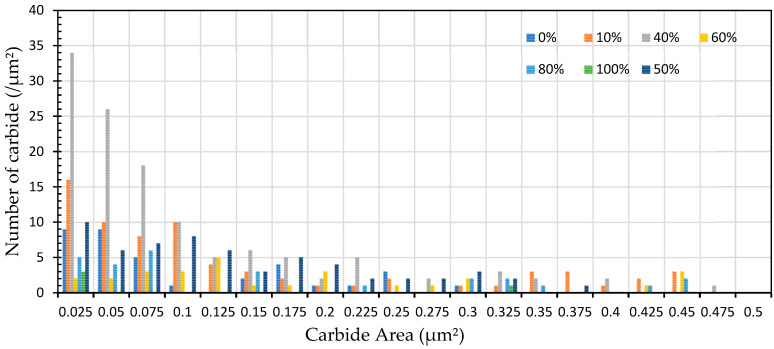
The distribution of carbide quantity (per µm^2^) vs. carbide area (µm^2^) for specimens subjected to heat treatment with varying spheroidized pearlite percentages (%).

The spheroidization ratio results obtained by Image J are presented in Figure 7 and Figure 8, which show that most of the lamellar pearlite, which consists of alternating layers of ferrite and cementite (carbide), was broken down into small carbide particles.

**Figure 9:**
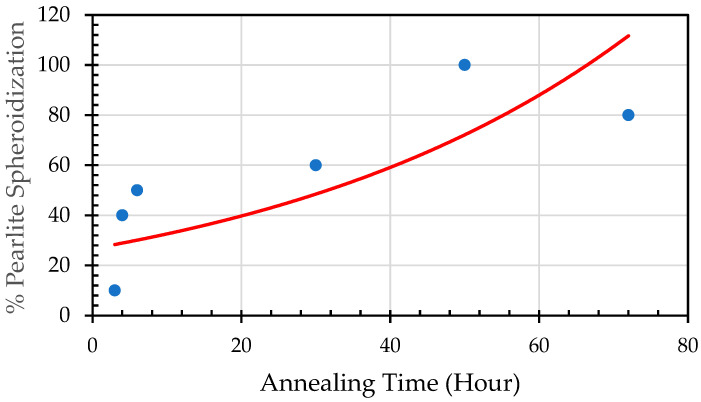
Percentage of spheroidized pearlite against annealing time.

By implementing image analysis, the distribution and percentage of spheroidized pearlite for each specimen can be obtained, allowing us to determine the correlation between heat treatment parameters and the percentage of spheroidized pearlite. Figure 2, Figure 3 and Figure 9 show that increasing the holding time during heat treatment leads to an increase in the number of lamellar pearlite particles transformed into spheroidized pearlite [12,13]. The effect of annealing time on the percentage of spheroidized pearlite can be seen in Figure 9.

This figure indicates that the longer the sample is exposed to heat in the furnace, the greater the percentage of spheroidized pearlite that can be obtained.

### 3.6. Texture Characterization of Spheroidized Pearlite Using Neutron Diffraction Method

Neutron diffraction is a powerful technique for characterizing the texture of spheroidized pearlite, offering detailed insights into the crystallographic orientation of the material. In this study, a texture neutron diffractometer (DN2) was installed in the experimental hall of the reactor (XHR) on beam tube S5 and operated at a neutron wavelength of 1.2799 angstroms, utilizing a helium (He) monitor as the detector and boron trifluoride (BF_3_) as the main detector [8,14].

The spheroidized pearlite sample was placed on an Euler cradle mounted on the sample table. Texture measurement involved tilting (χ) the sample within an angular range of 0° ≤ χ ≤ 90° and rotating (φ) it within 0° ≤ φ ≤ 360°. Data collection was carried out at a reactor power of 5 MW using the preset count (PRSC) method [15,16,17].

This analysis provides crucial information on the microstructural evolution of spheroidized pearlite, enhancing our understanding of its mechanical properties and potential applications in various industrial fields [18]. The spheroidizing process aims to transform carbide particles in steel from an irregular, elongated lamellar shape into a rounded (spheroid) form. Thus, spheroidized steel can be considered a heat-treatment (annealing)-induced modification of steel’s microstructure. This transformation significantly affects the particle size of the material, enhancing its ductility and machinability. In the crystal structure analysis, the spheroidized pearlite sample exhibits a body-centered cubic (BCC) crystal structure with a lattice parameter of 2.8665 angstroms [19,20]. In fact, ferrite itself has a BCC structure, but the overall crystallographic characteristics of lamellar pearlite can be complex due to interlamellar interfaces and stress states. The determination of the BCC structure of the spheroidized sample through neutron diffraction, as well as other characterization tests, indicates that the spheroidization process is a microstructural transformation rather than a fundamental change in the crystal structure. The pearlitic crystal structure does not change because, according to the Fe–C phase diagram, pearlite is stable in a BCC crystal structure at temperatures below 723 °C. Furthermore, the neutron diffraction measurements were conducted at room temperature; therefore, the crystal structure remains stable as BCC.

**Figure 10:**
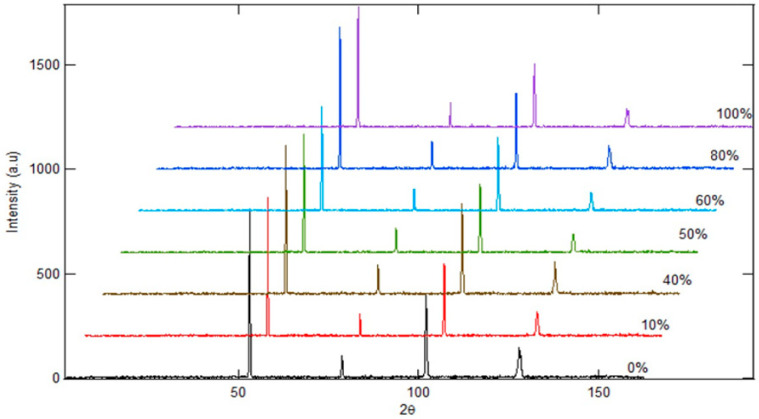
HRPD characterization of spheroidized pearlite using neutron beam.

Inspection was performed using high-resolution powder diffractometry (HRPD) with a neutron beam. The difference between this method and low-resolution powder diffractometry (LRPD) is related to the angular spacing of the measurements, with HRPD having a smaller spacing compared to LRPD. As a result, HRPD provides better data accuracy because overlapping peaks can be separated. In this study, the HRPD results indicate that the difference in the percentage of spheroidized pearlite affects the appearance of the Fe_3_C spectrum peak at the 2-theta angle [17,21,22]. The higher the percentage of spheroidized pearlite, the further the spectrum peak shifts to the right, appearing at a larger 2-theta angle (Figure 10).

Additionally, the percentage of spheroidized pearlite influences the intensity of the Fe_3_C spectrum, with a higher percentage of spheroidized pearlite resulting in lower spectrum intensity. This occurs because the atomic configuration in the lamellar form of Fe_3_C differs from the configuration in the spheroidal form [23,24].

### 3.7. X-Ray Diffractometry

**Figure 11:**
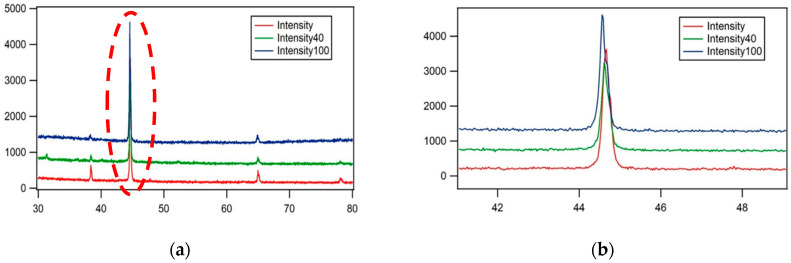
(**a**) The results of X-Ray Diffractometry (XRD) with observations at one of the spectrum angles, 2θ, in the range of 40–50. (**b**) The magnification of (**a**) at red dot area shows a shift in the spectrum peak.

XRD was conducted on test specimens heat-treated at each percentage of pearlite spheroidization, and the results are shown in Figure 11.

The results of the test specimen examination using an X-Ray Diffractometer (Figure 11) indicate peak shifts, peak broadening, intensity reduction, and the appearance of new peaks. These changes occur because pearlite spheroidization affects the atomic spacing in each structure, transitioning from a lamellar to a spheroidized form, which in turn influences the X-Ray Diffractometer spectrum configuration [25]. The alteration in atomic spacing within the bulk material may lead to a decline in mechanical properties, as dislocation movement within the material becomes easier under external forces, ultimately resulting in a decrease in tensile strength [26,27].

## 4. Discussion

### 4.1. Microstructural Evolution

The microstructures of the samples after heat treatment at particular times and temperatures were examined by optical and Scanning Electron Microscopy (SEM). Figure 2, Figure 3 and Figure 5 indicate that spheroidization of pearlite is highly affected by annealing time and temperature. The longer a sample is exposed to heat in the furnace, the greater the percentage of spheroidized pearlite that can be obtained (Figure 9). This means that the longer boiler tubes are exposed to high temperatures during operation, the higher the percentage of spheroidized pearlite formed. The experimental results indicate that increasing the time of exposure (from 30 h to 72 h) has a greater effect than increasing the temperature from 650 °C to 750 °C (Figure 2 and Figure 3). The distribution of spheroidized particle sizes between 0 and 6 h of annealing, shown in Figure 7 and Figure 8, indicates that the largest group of spheroidized particles is below 0.225 µm^2^. These results suggest that the increase in spheroidized particles is due to the fragmentation of lamellar cementite, which occurs at the initial stage of annealing to reduce surface energy [12,13]. As a result of this process, the fractured cementite rapidly transforms into a spherical shape. Specifically, the area fraction of spheroidized cementite with a size of 0.425 µm^2^ is higher at an 80% spheroidized pearlite percentage. There is little change in microstructure between samples annealed at spheroidized pearlite percentages of 40% and 50%, where, during spheroidization, the newly formed particles develop at the same rate as the coarsening of the pre-existing particles [8,14]. The distribution at 100% spheroidization shows that spheroidized cementite particles of 0.325 µm^2^ exhibit a greater increase compared to newly formed spheroidized particles (0.025 µm^2^). This occurs because with longer annealing time, the spheroidized particles tend to cluster (agglomerate), resulting in larger spheroidized particles [9]. However, at 100% spheroidization, the spheroidized particles remain below 0.35 µm^2^. Figure 7 and Figure 8 show that approximately 131 carbide particles per µm^2^ undergo spheroidization, indicating that pearlite fragmentation occurs under these conditions. Additionally, the number of carbide particles decreases to 31 particles/μm^2^ after 72 h of heat treatment at 700 °C, while pearlite fragmentation continues up to 50 h during heat treatment at 650 °C in the spheroidization process [15,16].

### 4.2. Small-Angle X-Ray Scattering (SAXS) Analysis

**Figure 12:**
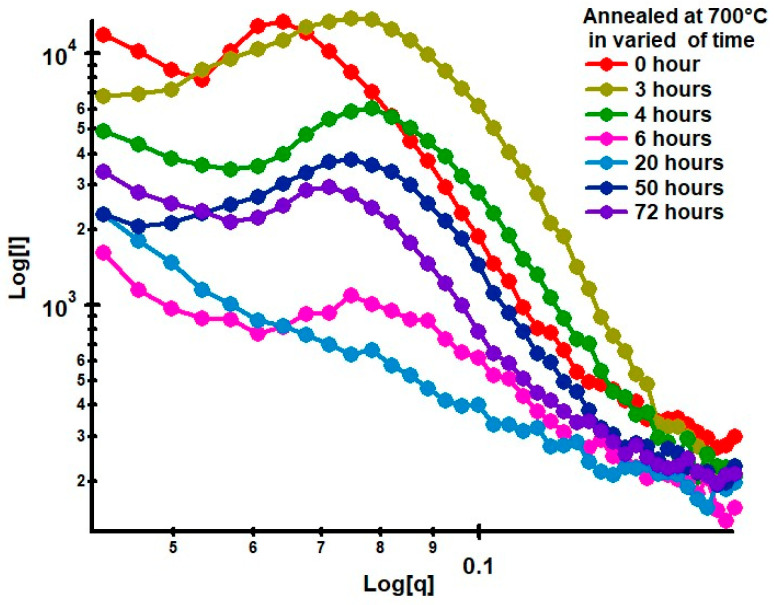
SAXS patterns of steel with 0.32% carbon content after annealing at 700 °C for 0, 3, 4, 5, 20, 50, and 72 h.

Small-Angle X-ray Scattering (SAXS) is a widely used technique for analyzing microstructural changes in materials, especially those related to morphological transformations caused by thermal treatment. Researchers have also conducted quantitative analyses of the spheroidization process in the pearlite system using small-angle scattering techniques with neutrons as the probe. In this study, the spheroidization of cementite in pearlite was analyzed using the SAXS technique for steel with a low carbon content of about 0.184%. The carbon steel was then annealed at a temperature of 700 °C for durations of 0, 3, 4, 6, 20, 50, and 72 h. The X-ray scattering data from the log I(q) vs. log q(Å^−1^) measurements are presented in Figure 12.

**Figure 13:**
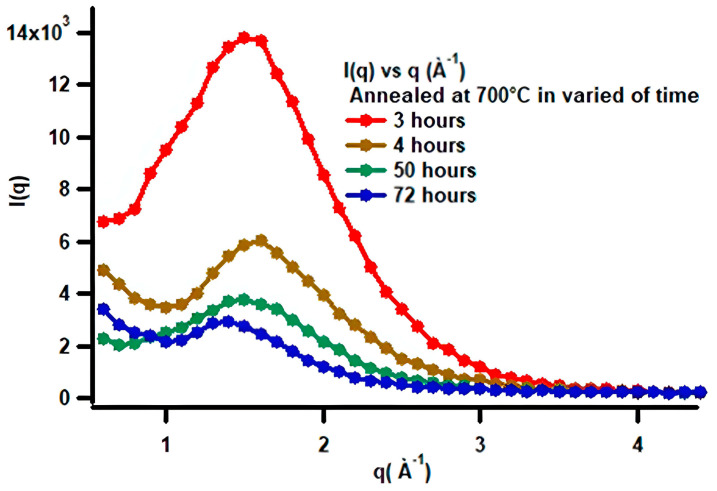
SAXS patterns of heat-treated low-carbon steel after annealing at 700 °C for 3, 4, 50, and 72 h.

To observe the effect of heat treatment on low-carbon steel, especially in relation to morphological changes during spheroidization, one can examine the changes in scattering intensity as a function of annealing time. Figure 13 shows the SAXS characterization results obtained in this study for annealing durations of 3, 4, 50, and 72 h.

Figure 13 shows that the SAXS patterns exhibit a shift in the scattering peak position toward smaller q values as the annealing time increases. Considering the relationship d = 4π sin(θ)/λ, for small-angle scattering, this can be approximated as d = 2π/q, which indicates that atomic diffusion into the core not only enlarges (coarsens) the particles but also increases their spatial separation. This is consistent with the SEM observations, which show that with increasing annealing time, particle clustering becomes evident. The decrease in intensity with increasing annealing time is likely related to the reduction in phase contrast due to accelerated atomic diffusion during heat treatment. In other words, the shift in the peak toward lower q values and the decrease in intensity are caused by microstructural reorganization within the low-carbon steel system. A more quantitative analysis was carried out using the measured data, processed with SASView software version 6.0.

### 4.3. Quantitative Analysis of SAXS Data

**Figure 14:**
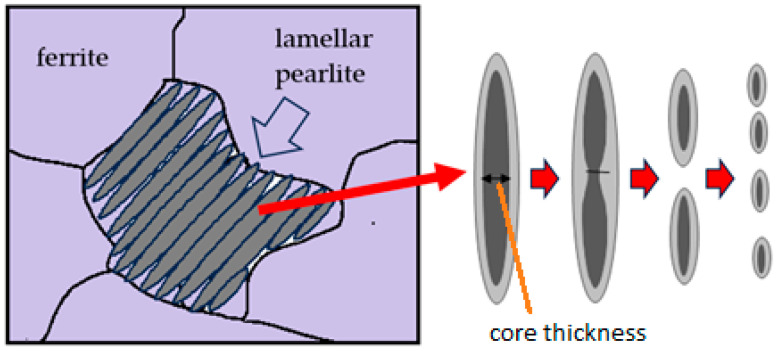
The schematic mechanism of transformation from lamellar pearlite into spheroidized pearlite/cementite.

Quantitative analysis of the SAXS data was carried out using SASView software version 6.0. A PANalytical diffractometer was used for the SAXS measurements, employing X-ray scattering with a Cu-Kα target at room temperature. Prior to the measurements, the sample surface was polished using 1000-grit sandpaper. Based on the SEM observations, the microstructural deformation process of the heat-treated low-carbon steel transitions from a lamellar system to a spheroidized system, depending on the annealing duration. The microstructural changes were analyzed using the Small-Angle X-ray Scattering method. Considering that an ellipsoidal ferrite structure surrounded by an Fe_3_C shell is likely to form in a low-carbon steel system, the SAXS analysis employed the “ellipsoid-core model” [28] and is presented in Figure 14.

**Figure 15:**
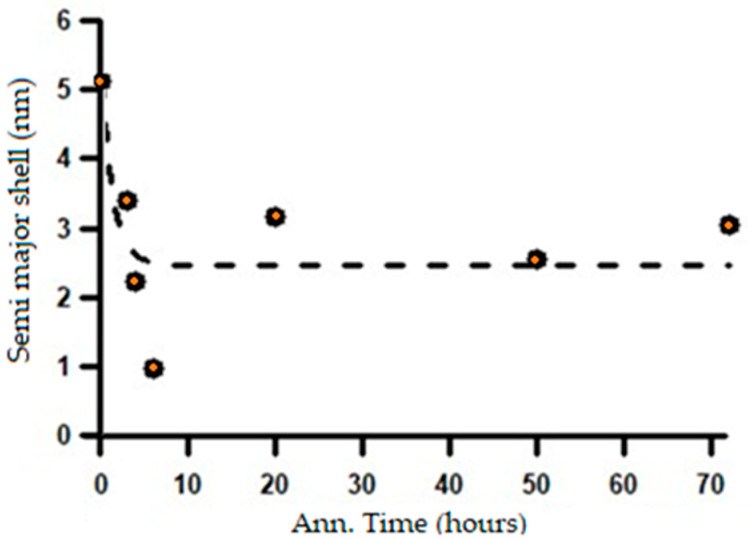
The change in the length of the major axis with increasing annealing time.

**Figure 16:**
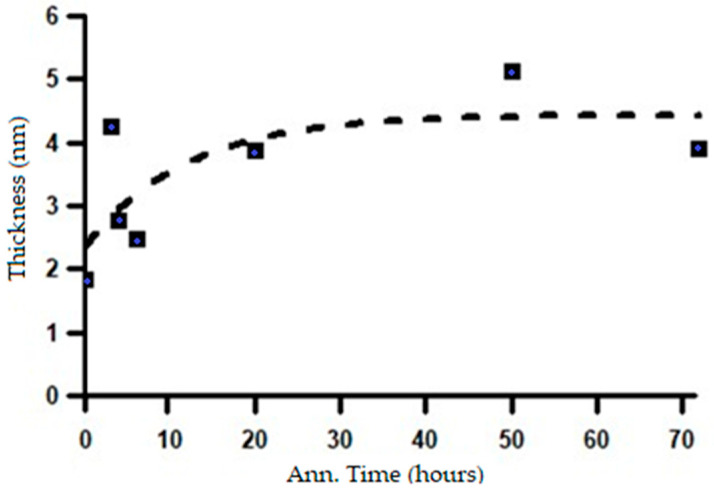
The change in shell thickness with increasing annealing time.

Based on the fitting results shown in Figure 15, it is evident that, under the initial condition prior to annealing, the material exhibits a very long major axis. This observation is consistent with the microstructure consisting of thin lamellar layers, as confirmed by SEM observations in Figure 4. However, after heat treatment, the major axis of the ellipsoid decreases drastically and then tends to stabilize with further annealing. This reduction in the major-axis length with increasing annealing time is closely associated with the microstructural transformation from a lamellar morphology to spheroidized particles. This process is likely accompanied by phase redistribution, which may involve carbon diffusion, carbide redistribution, or other segregation phenomena. Initially, these features appear as large domains that gradually diffuse into the matrix through thermal diffusion. This interpretation is in good agreement with the SEM observations shown in Figure 5a,b. Morphological stability is achieved after approximately 20–30 h of annealing, as indicated by the results in Figure 15. This interpretation is further supported by the analysis of the shell thickness, which increases with increasing annealing time, as shown in Figure 16. These results are in very good agreement with the SANS observations reported by Su Y. et al. [17] on the spheroidization process in carbon steel. The transformation from lamellar pearlite to spheroidized pearlite does not involve the formation of a new phase. Both microstructures consist of ferrite (α-Fe) and cementite (Fe_3_C); however, spheroidization is characterized by a morphological change in cementite from lamellar plates to spheroidal particles, driven by interfacial energy minimization. No distinct carbon-rich phase or interfacial transition layer is formed, although local carbon gradients may exist near the ferrite–cementite interface during the transformation process.

**Figure 17:**
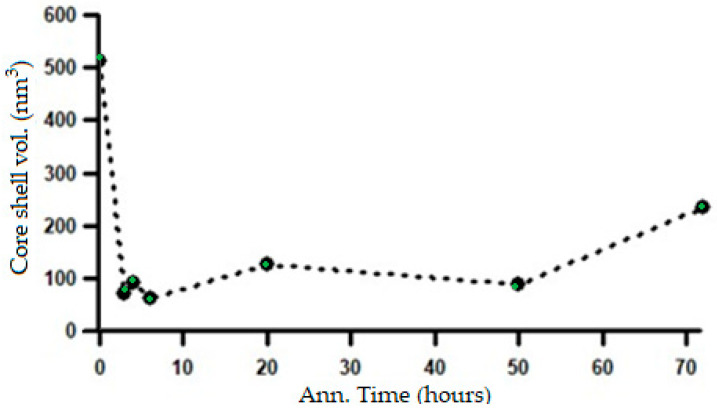
Change in shell volume with increasing annealing time based on SAXS analysis using the ellipsoid core–shell model.

The changes in the length of the major axis and the shell thickness suggest that the microstructural transformation from a lamellar system to a spheroidized system begins during the initial stages of heat treatment at 700 °C and continues throughout the annealing process. The microstructural changes corresponding to the variations in the SAXS intensity patterns, shown in Figure 13, can be confirmed by the changes in the shell volume in relation to the heating duration, as shown in Figure 17.

### 4.4. Evolution of Mechanical Properties

#### 4.4.1. Evolution of Hardness Values

**Figure 18:**
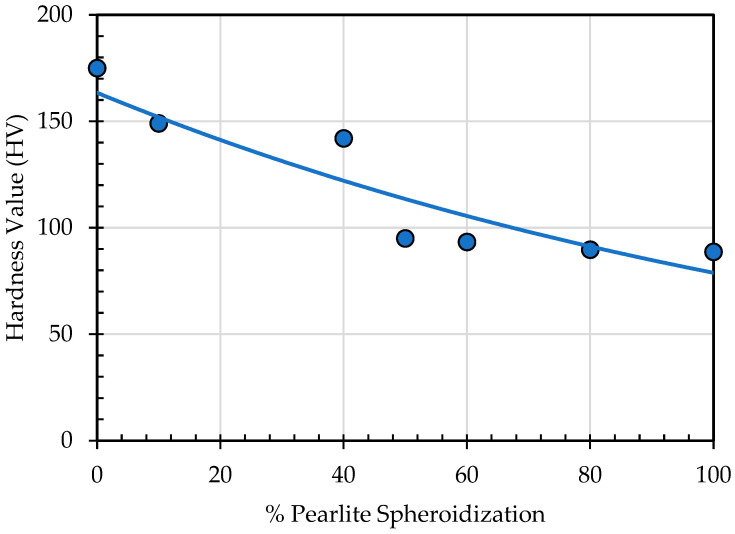
Effect of spheroidized pearlite percentage on hardness value.

A hardness test was conducted on specimens with different percentages of spheroidized pearlite, and the results are presented in Table 4 and Figure 18. It is shown that the hardness value decreases as the percentage of spheroidized pearlite increases. This phenomenon occurs because, by increasing annealing time, all of the internal stress can be alleviated, and the grains damaged by previous mechanical treatment or machining can be recrystallized. Moreover, some of the dislocation pile-up caused by cold working or other types of cold deformation is eradicated with longer annealing time, thus decreasing the hardness value.

#### 4.4.2. Strength at Elevated Temperatures

**Figure 19:**
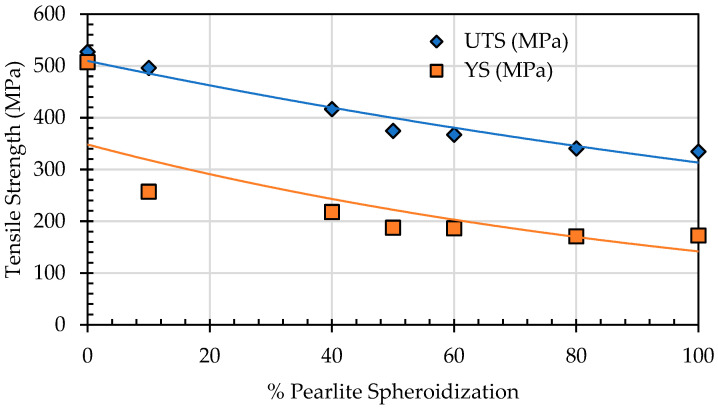
Effect of spheroidized pearlite percentage on tensile strength.

To evaluate the changes in the mechanical properties of spheroidized pearlite specimens of SA 178 steel at elevated temperatures, a tensile test was conducted at an operational temperature of 275 °C. The test results indicate that as the percentage of spheroidized pearlite increases, the tensile strength decreases (Figure 19).

This occurs because, under tensile loading, dislocations move more easily in the spheroidized pearlite microstructure compared to the lamellar structure [29]. In pearlitic steels, cementite (Fe_3_C) lamellae act as critical barriers to dislocation motion, governing the material’s high strength and hardening behavior. The interaction between dislocations in the ductile ferrite phase and the brittle cementite lamellae creates several complex dynamics [9]:

1. Obstacle and Boundary Strengthening
  (a) Barriers to Glide: Cementite lamellae function similarly to grain boundaries, strongly resisting dislocation glide. This forces dislocations to pile up at the ferrite–cementite interface, leading to significant stress concentrations.
  (b) Mean Free Path Limitation: The interlamellar spacing (ILS) defines the “mean free path” for dislocations. Reducing this spacing significantly enhances yield stress—often described by a modified Hall-Petch relationship—as dislocations have less room to move and multiply.
2. Interface Interactions
  (a) Sources and Sinks: The ferrite–cementite interface serves as both a source for nucleating new dislocations and a “sink” where existing dislocations can be trapped or annihilated.
  (b) Dislocation Trapping: At very small scales (nanoscale), the interface can absorb lattice dislocations through “image forces” and interfacial shear, which helps manage local stress.

**Figure 20:**
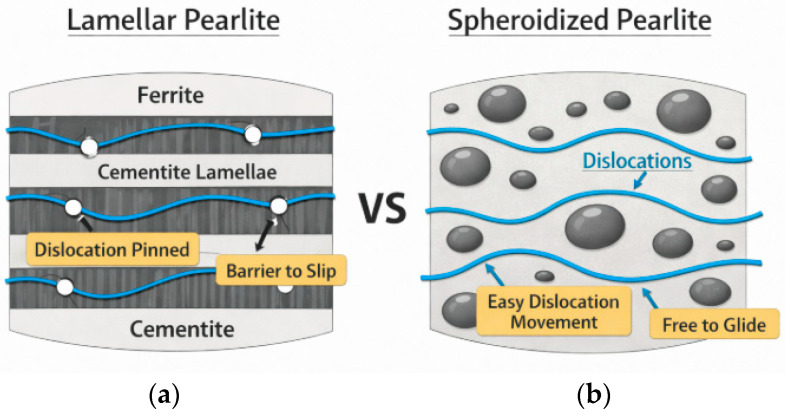
Schematic figure of dislocation movement on (**a**) lamellar pearlite where dislocations are pinned by cementite lamellae, and (**b**) dislocations glide through ferrite matrix.

A comparison of dislocation motion between lamellar pearlite and spheroidized pearlite is shown in Figure 20.

**Figure 21:**
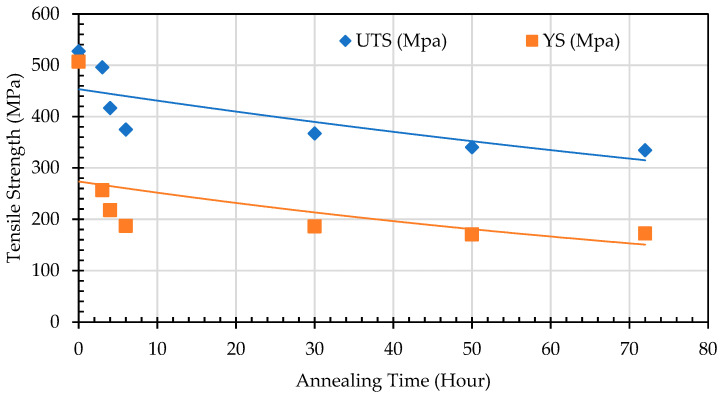
Effect of annealing time on tensile strength of SA 178 materials.

In lamellar pearlite, dislocations are restricted by the close spacing of cementite plates, which act as formidable barriers. Once spheroidized, the mean free path for dislocation movement within the continuous ferrite matrix increases significantly. Dislocations can glide over much longer distances before encountering a cementite particle, leading to a reduction in tensile strength [29]. The analysis of the effect of annealing time on the mechanical properties of SA 178 shows that increasing annealing time leads to a decrease in tensile strength (Figure 21).

**Figure 22:**
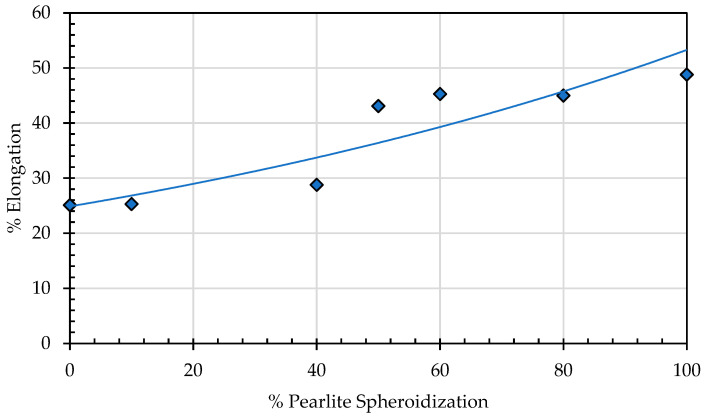
Effect of pearlite spheroidization on elongation of SA 178 materials.

This phenomenon occurs due to several factors, including the increasing percentage of spheroidized pearlite and the reduction in obstacles from deformed grains that have undergone recrystallization. These two factors facilitate dislocation movement under mechanical loading, leading to a decrease in tensile strength and increasing elongation (Figure 22). The lamellar pearlite structure presents greater obstacles to dislocation movement, whereas the spheroidized structure allows for easier dislocation motion, resulting in improved ductility of the metal [30,31].

**Figure 23:**
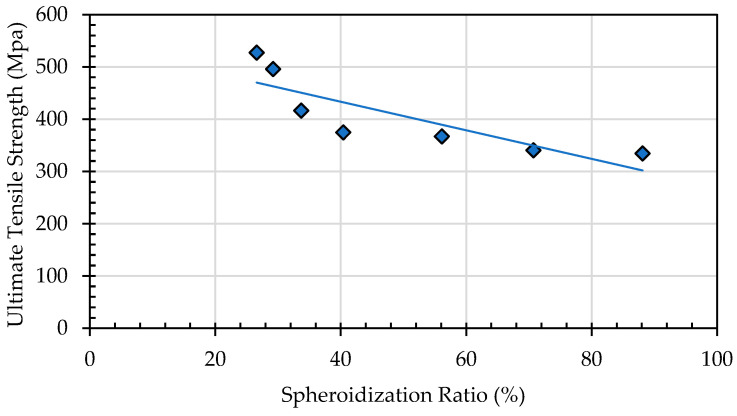
Effect of spheroidization ratio (%) on ultimate tensile strength (MPa).

Annealing time significantly affects the elongation properties of the material, as a longer annealing duration means more thermal energy is applied to the test specimen. This not only facilitates the transformation of lamellar pearlite into a spheroidized structure, but also promotes recrystallization and the rearrangement of atoms into a more ordered configuration. As a result, resistance to dislocation movement decreases, making the material more capable of flowing in the direction of the applied tensile load [32,33]. Consequently, elongation increases with longer annealing time. The spheroidization ratio, calculated as the proportion of the spheroidized pearlite area to the total area, indicates that a higher spheroidization ratio corresponds to lower tensile strength. This occurs because the greater the amount of lamellar pearlite that undergoes spheroidization within a given area, the easier it becomes for dislocations to move under tensile loading compared to the lamellar pearlite structure (Figure 23).

Spheroidization of SA-178 Grade C significantly affects fracture toughness and the ductile-to-brittle transition temperature. The transformation of lamellar cementite into spheroidal particles reduces stress concentration and suppresses cleavage crack initiation, leading to increased impact toughness and a shift in the ductile-to-brittle transition temperature (DBTT) toward lower temperatures. Although boiler tubes typically operate at temperatures well above room temperature, the ductile-to-brittle transition temperature remains relevant for safety assessment during non-steady conditions such as cold start-up, shutdown, hydrostatic testing, and impact or dynamic loading. Microstructural evolution associated with pearlite spheroidization can shift the DBTT to lower temperatures, thereby improving fracture resistance under these critical conditions.

## 5. Conclusions

Based on the results of the experiments conducted in this study and the in-depth analysis of SA 178 as a boiler material, the following can be concluded:

▪ The transformation of lamellar pearlite into spheroidized pearlite is initiated by a change in the very long major axis of the ellipsoidal structure, which is characteristic of a microstructure composed of thin lamellar layers. Subsequently, the major axis decreases drastically, accompanied by an increase in shell thickness, leading to the separation of individual lamellae and their evolution into a spheroidized form.
▪ The number of spheroidized pearlite particles increases with an increasing duration of exposure to elevated temperatures.
▪ A higher percentage of spheroidized pearlite decreases the tensile strength and hardness of the boiler tube material.
▪ A decrease in tensile strength and hardness reduces that material’s ability to withstand operational loads.

Therefore, if the operating temperature of a boiler remains constant between 250 °C and 350 °C, the remaining service life of the boiler tubes can be estimated by monitoring the percentage of spheroidized pearlite.

## Figures and Tables

**Table 1:** Chemical Composition of AISI SA 178 grade C carbon steel.

Element (wt%)	C	Mn	P	S	Fe
Standard specification	0.35 Max.	0.80 Max.	0.035 Max.	0.035 Max.	Bal.
Test result	0.184	0.452	0.0138	0.0051	Bal.

**Table 2:** Temperature and time parameters for heat treatment to obtain spheroidized pearlite.

Heating Temperature	Holding Time (Hours)				
650 °C	4	6	30	50	72
750 °C	3	6	30	50	-
Cooling	All of specimens are slow cooled (furnace cooling)				

**Table 3:** EDX spectrum of particles that formed after heat treatment.

Element	(wt%)
Fe	67.55 ± 1.40
C	32.45 ± 0.39

**Table 4:** Vickers hardness test results.

Spheridized Pearlite (%)	0	10	40	50	60	80	100
Hardness Value (HV)	175	149	142	95	93	89	88

## Data Availability

The original contributions presented in this study are included in the article. Further inquiries can be directed to the corresponding author.

## References

[1] Suhadi A., Febriyanti E., Sari L.N. (2021). The Role of Failure Analysis on Maintaining Reliability of Oil Refinery for Sustainable Development Goals. IOP Conf. Ser. Mater. Sci. Eng..

[2] Chatterjee U.K. (2012). Microstructural imprints in failure of power plant boiler tubes. Int. Conf. Appl. Mech. Mech. Eng..

[3] Suhadi A., Aprilio A., Febriyanti E. (2023). Structural Strength Degradation of Oil and Gas Refinery Equipment. Case Study: Heat Exchanger Tubes of Hydrocarbon Vapor. Evergreen.

[4] Syahril M., Suhadi A., Febriyanti E., Afandi Y., Karuana F. (2024). Evaluation of refinery unit tube heater condition after ±15 years in service by NDT methods. AIP Conf. Proc..

[5] Zhao Q.-H., Jiang B., Wang J.-M. (2016). Pearlite Spheroidization Mechanism and Lifetime Prediction of 12Cr1MoV Steel used in Power Plant. Proceedings of the 4th 2016 International Conference on Material Science and Engineering.

[6] ASM Handbook Committee (2005). Properties and Selection: Irons, Steels, and High Performance Alloys. ASM Handbook.

[7] Felipe G.B.U., Junca E., Arnt Â.B.C., da Rocha M.R., Dal-Bó A.G. (2020). Heat treatment analysis of astm a106 steel spheroidization and erosive wear at high temperatures. REM—Int. Eng. J..

[8] Joo H.S., Hwang S.K., Baek H.M., Im Y.-T., Son I.-H., Bae C.M. (2015). The effect of a non-circular drawing sequence on spheroidization of medium carbon steel wires. J. Mech. Work. Technol..

[9] Alza V.A. (2021). Spheroidizing in Steels: Processes, Mechanisms, Kinetic and Microstructure—A Review. IOSR J. Mech. Civ. Eng..

[10] Song W., Choi P.-P., Inden G., Prahl U., Raabe D., Bleck W. (2013). On the spheroidized carbide dissolution and elemental partitioning in a high carbon bearing steel 100Cr6. Met. Mater. Trans. A.

[11] Czarski A., Skowronek T., Matusiewicz P. (2015). Stability of a lamellar structure—Effect of the true interlamellar spacing on the durability of a pearlite colony. Arch. Met. Mater..

[12] Amos P.K., Bhattacharya A., Nestler B., Ankit K. (2018). Mechanisms of pearlite spheroidization: Insights from 3D phase-field simulations. Acta Mater..

[13] Bhadeshia H.K.D.H. (2021). Theory of Transformations in Steels.

[14] Nutal N., Gommes C.J., Blacher S., Pouteau P., Pirard J.-P., Boschini F., Traina K., Cloots R. (2010). Image analysis of pearlite spheroidization based on the morphological characterization of cementite particles. Image Anal. Ster..

[15] Wang S., Cao L., Zhang Z. (2019). Influence of carbide morphology on the deformation and fracture mechanisms of spheroidized 14CrMoR steel. Metals.

[16] O’bRien J.M., Hosford W.F. (2002). Spheroidization Cycles for Medium Carbon Steels. Met. Mater. Trans. A.

[17] Su Y., Morooka S., Ohnuma M., Suzuki J., Tomota Y. (2015). Quantitative Analysis of Cementite Spheroidization in Pearlite by Small-Angle Neutron Scattering. Met. Mater. Trans. A.

[18] Tomota Y., Wang Y.X., Ohmura T., Sekido N., Harjo S., Kawasaki T., Gong W., Taniyama A. (2018). In situ neutron diffraction study on ferrite and pearlite transformations for a 1.5Mn-1.5Si-0.2C steel. ISIJ Int..

[19] Tejero-Martin D., Bai M., Mata J., Hussain T. (2021). Evolution of porosity in suspension thermal sprayed YSZ thermal barrier coatings through neutron scattering and image analysis techniques. J. Eur. Ceram. Soc..

[20] Onuki Y., Hirano T., Hoshikawa A., Sato S., Tomida T. (2019). In Situ Observation of Bainite Transformation and Simultaneous Carbon Enrichment in Austenite in Low-Alloyed TRIP Steel Using Time-of-Flight Neutron Diffraction Techniques. Met. Mater. Trans. A.

[21] Gray V., Galvin D., Sun L., Gilbert E., Martin T., Hill P., Rawson M., Perkins K. (2017). Precipitation in a novel maraging steel F1E: A study of austenitization and aging using small angle neutron scattering. Mater. Charact..

[22] Chung P.P., Mata J., Wang J., Durandet Y. (2024). Application of Small- and Ultra-Small-Angle Neutron Scattering for the Characterization of Mechanically Plated Coatings. J. Mater. Eng. Perform..

[23] Ji Y., Radlinski A.P., Blach T., de Campo L., Vu P., Roshan H., Regenauer-Lieb K. (2022). How to avoid multiple scattering in strongly scattering SANS and USANS samples. Fuel.

[24] Barré L. (2015). Contribution of small-angle X-ray and neutron scattering (SAXS and SANS) to the characterization of natural nanomaterials. X-Ray and Neutron Techniques for Nanomaterials Characterization.

[25] Gräwert M., Svergun D. (2020). A beginner’s guide to solution small-angle X-ray scattering (SAXS). Biochemist.

[26] Simm T.H., Sun L., Galvin D.R., Hill P., Rawson M., Birosca S., Gilbert E.P., Bhadeshia H., Perkins K. (2017). The effect of a two-stage heat-treatment on the microstructural and mechanical properties of a maraging steel. Materials.

[27] Shin E., Seong B., Han Y., Lee C., Kim H. (2004). Study of precipitate and texture in low-carbon steels by neutron scattering techniques. Phys. B Condens. Matter.

[28] (2025). Nist Igor/Danse, and Steve King. core_shell_ellipsoid. https://www.sasview.org/docs/user/models/core_shell_ellipsoid.html.

[29] Seong B.S., Cho Y.R., Shin E.J., Kim S.I., Choi S.-H., Kim H.R., Kim Y.J. (2008). Study of the effect of nano-sized pre-cipitates on the mechanical properties of boron-added low-carbon steels by neutron scattering techniques. J. Appl. Crystallogr..

[30] Mates S., Stoudt M., Gangireddy S. (2016). Measuring the Influence of Pearlite Dissolution on the Transient Dynamic Strength of Rapidly Heated Plain Carbon Steels. JOM.

[31] Jia N., Shen Y.F., Liang J.W., Feng X.W., Wang H.B., Misra R.D.K. (2017). Nanoscale spheroidized cementite induced ultrahigh strength-ductility combination in innovatively processed ultrafine-grained low alloy medium-carbon steel. Sci. Rep..

[32] Chen J., Jin S.-J., Qiao D. (2020). Nondestructive evaluation of microstructure degradation of pearlitic steel. Proceedings of the 2020 IEEE Far East NDT New Technology and Application Forum, FENDT 2020.

[33] Grygier D., Dudziński W., Gerstein G., Nürnberger F. (2015). The effectiveness of spheroidization pearlitic steel with regard to the degree of plastic deformation. Interdiscip. J. Eng. Sci..

